# Risk and Safety Management in Physical Education: A Study of Teachers' Practice Perspectives

**DOI:** 10.3389/fspor.2021.663676

**Published:** 2021-04-16

**Authors:** Lise Porsanger, Leif Inge Magnussen

**Affiliations:** ^1^Department of Teacher Education, Norwegian University of Science and Technology, Trondheim, Norway; ^2^Department of Business, History and Social Sciences, University of Southeastern Norway, Borre, Norway

**Keywords:** risk management, safety, pedagogy, teacher practice, physical education

## Abstract

The aim of this article is to explore and understand teachers' risk and safety management (RSM) practices in physical education (PE) programs in Norway. A survey questionnaire and semistructured interviews were therefore used to generate quantitative data on trends from a larger sample of teachers (*n* = 698) and rich in-depth qualitative data concerning teachers' (*n* = 17) practices. By providing the teachers' perspectives, a better understanding of the complexity of RSM in PE may be possible. The results from both the survey and interviews suggest that teachers employ multiple strategies: from safety procedures, complying to compulsory risk measures, to the use of common sense in their RSM practices. The interviewees, on the other hand, initially claim that their RSM practice is quite scarce and, in some respects, not appropriate for PE. They emphasize measures that cater to the students' needs and modification to physical activities in their teaching. However, the interview data suggest that teachers do not primarily conceptualize this part of their practice as RSM but as measures of other pedagogical concerns. Combined, the results from both the survey and the interviews may characterize a RSM practice that relies on teaching experience and the use of discretion. The results in this article both converge and diverge and emphasize the importance of multiple data sources in investigating teachers' RSM practices.

## Introduction

The inherent element of bodily movement in school physical education (PE) programs generates a risk for accidents and physical injury to students. The ways teachers practice risk and safety management (RSM) in PE clearly inflict on students' educative opportunities. In the UK, for example, former school safety policies and teachers' fear of liability restricted children's development; educators were therefore given an updated mandate to balance educational risk through risk–benefit assessments and the use of common sense (HM Government, 2010). However, there seems to be a paucity of empirical studies that have investigated how teachers practice RSM in PE programs and, in particular, from the perspective of teachers (Park, 2018). Some related empirical studies investigate how teachers practice RSM in selected sports such as floor hockey (Gray, 1992) or why teachers are hesitant to teach gymnastics (Robinson et al., 2020) while others seem oriented toward teachers' liability concerns in practice (Young, 2007; Rothe, 2009). The current research-based knowledge remains scarce, and this article therefore seeks to explore and understand teachers' RSM practices in PE teaching, and a survey questionnaire and interviews with teachers are conducted to collect data.

Advice and instructions for teachers' practices are based on identified hazards (Podstawski et al., 2015) or scenarios that may evolve in PE classes (Merrie et al., 2016). To prevent student injuries and protect teachers from liability, an additional strand of RSM instructions stems from the analysis of tort law and teaching negligence cases (Murphy and Beh, 2014; Gimbert and Sawyer, 2015; McCoy et al., 2017). Relating to best-practice risk management that is presented in sports (Fuller and Drawer, 2004), the use of safety guidelines and rules seems to be a central strategy for safe teaching practices in PE. Some might adhere to safety principles described in primary school PE literature for example (e.g., Severs et al., 2003). There are, however, some disagreements in the field. While Rothe (2009) suggests that safety guidelines ought to be voluntary for teachers, others point to peer observation, and checks to ensure standards and guidelines are appropriately implemented (Fitzgerald and Deutsch, 2016). Based on these points, the literature seems oriented toward how teachers may reduce, control, or eliminate risk to prevent accidents and injuries through prescribed procedures.

At the same time, PE teachers are also called out to embrace the exploration and uncertainty of transformative teaching practices (Quennerstedt, 2019). Considering the literature on RSM in PE, it seems to contrast the call for transformative pedagogy. Albeit it may be that the pedagogical focus is on learning and not risk (Brown and Fraser, 2009), educational pedagogy may, in some respects, be “romancing risk” and position educators in crucial dilemmas and maybe even to rely on luck, which is indicated in adventure education (Bell, 2017, p. 284). However, there is also worry that in situations where concerns for liability are prominent, professionals might rule out their experience-based expertise and rely on procedures to protect themselves (Zinn, 2016). In outdoor and adventure education, some suggest that rational discourses and methodology surrounding RSM might restrain practitioners' subjective judgements and experience (Zink and Leberman, 2001). It therefore seems plausible that risk aversion in education might restrict students of educative opportunities (Biesta, 2013). In addition, strategies of insurance and assurance may come to prevail teachers' practices if accountability is stressed (Lindqvist et al., 2009). In Norwegian PE, the language in a regulative orientation on how to teach water activities in schools might give an impression of being compulsory and therefore restrict PE teachers' RSM practices to the use of recommendations (Porsanger, 2020). Again, Korean primary school PE teachers' fear of litigation might induce them to exclude certain activities from PE (Park, 2018). However, the primary reason for risk aversion in a Canadian study was reported to be concerns for students' safety and not necessarily litigation (Young, 2007). Upper secondary school teachers in Norway also exclude students from outdoor education excursions due to safety concerns (Dahl et al., 2019).

Teachers, as well as other professions, must deal with dilemmas in their practices, and in PE, they might have to both embrace and reduce risk to generate safe learning environments and educative opportunities for students. In outdoor adventure, the aspects of balance and paradox in risk management is not new (e.g., Collins and Collins, 2013). Martínková and Parry (2017) suggest that educators may employ “Safe Danger” to open adventure experiences to students but not cause severe harm. However, there seems to be a paucity of empirical investigations of how teachers' balance risk in PE. In the UK, there are indications that Forest School educators still experience tension between their pedagogies and societal risk aversion (Connolly and Haughton, 2017). Risk research suggests that different values and perspectives on risk might generate ambiguity (Aven and Renn, 2020). The balance of educational risk might be tricky for teachers due to dilemmas between multiple concerns in their teaching. PE teachers' RSM practices might be comparable to those of other professions with “risk work” characterized by multiple concerns and strategies (Brown and Gale, 2018a,b). The dynamic and complex environments of outdoor instructors seem to require adaptive expertise (Mees et al., 2020), and it seems plausible that the environment of PE teaching might require teachers to be equally flexible. Due to the scarcity of studies that have investigated teachers' RSM practices, this article therefore makes use of a risk strategy typology developed by Zinn (2016) in a discussion of the results: to differentiate between teachers' risk strategies and suggest how they combine them in their practice. A brief account of the typology and how it may be relevant in teaching is presented next.

### Combining Risk Strategies in Teaching Physical Education

There is potential to extend the current understanding of RSM practices in PE by conferring Zinn's (2016) risk strategy typology, and in this article, the typology is used to discuss the results. Primarily, the typology differentiates between three types of risk strategy: rational, in-between, and non-rational risk strategies (Zinn, 2016), and it may therefore assist to better understand teachers' different approaches to risk and their rationale. However, risk practices are embedded within a political and cultural environment (Lupton, 2013; Zinn, 2019) and not mere aggregates of individual choice (Douglas, 1992). Another strength of the typology is therefore in the multiple combinations of these strategies to create “reasonable” practices depending on the actors' social context (Zinn, 2016). The Norwegian PE teachers' RSM practices might be customized based on the national curricular requirements and risk policy as well as on local school arrangements.

#### Rational Strategies

Rational risk strategies derive from an instrumental reasoning of “direct management and control of risk” (Zinn, 2016, p. 351). They might therefore be rational for professions, recognized for their specialized knowledge and technical skills anchored in a scientific and theoretical knowledge base (Freidson, 2001). Assuming that some risks may be predictable and controllable (Renn, 2008), general or abstract principles might be pulled out and applied to address risk and uncertainty in PE. Hence, applying the correct method might be expected to solve the problem in some respects (Schön, 1995). Teachers may deliberately use risk matrices and checklists to assess and determine courses of action in their teaching and might be necessary for teachers to grasp risk in PE because “the transformation of uncertainty into probability enables us to deal with uncertainty as if we had knowledge” (Merkelsen, 2011). There are thus reports on teachers using safety guidelines in their PE teaching for example (Rothe, 2009). However, the degree of potential certainty is contended (Aven and Renn, 2020), and in PE, it might be complicated to calculate or foresee the actions of students or every outcome of sports and play. A dilemma of risk analysis also relates to the ratings of likelihood and consequence, as they might be subject to a high degree of variation and merely subjective guesses (Cox, 2008). Thus, rational strategies might be appropriate for some conditions or elements in PE where causes and outcomes may be accessible for teachers, while other aspects of risk might be overlooked or disregarded. The in-between strategies may therefore complement rational strategies in teachers' RSM practice: addressing other dimensions and concerns with risk in PE.

#### In-between Strategies

In-between strategies seem to be characteristic of the situational and practical reasoning of professionals' “risk work” (Horlick-Jones, 2005). Drawing on experience, teachers' tacit knowing (Polanyi, 1983) and knowing-in-action (Schön, 1995) might enhance the use of in-between strategies in PE. As a result, there might not be any explicit methods or systems thinking behind teachers' risk judgments in practice but ongoing reasoning and action characteristics of professional discretion (Freidson, 2001). As feelings and affect may guide actors' risk decisions (Lupton, 2013), emotions, intuition, and trust might be just as reasonable in dealing with risk and uncertainty in some respects (Zinn, 2016). Teachers' concerns in PE might generate common RSM practices; for example, the fear of adverse consequences, students' safety (Young, 2007), or liability (Park, 2018) might lead to risk-averse teaching practices in PE. However, in-between and non-rational strategies are often used in combination (Zinn, 2016) and might be crucial for teachers in balancing their RSM practice.

#### Non-rational Strategies

The use of non-rational strategies relies more on attitude than knowledge (Zinn, 2016), and hope, ideology, and belief might complement the other risk strategies in teachers' practices. However, risk in PE is not necessarily equal to danger but also resonates with an adventure concept and is framed as an opportunity for learning, as uncertainty might generate educative experiences (Quennerstedt, 2019). While the risk strategy typology seems to focus on managing adverse consequences, teachers' non-rational strategies might also draw on the risk benefits of actively engaging with risk to enhance something. Thus, teachers' RSM practices might contain an element of active risk-taking or making (Zinn, 2019) and a more passive acceptance of risk due to lack of means (Zinn, 2016).

However, “the key question pertains to the skills and experience one needs to decide about the appropriate combination of strategies to use in a particular situation” (Zinn, 2016, p. 361). This aspect of practice might be contingent on how teachers' institutional environments support the different approaches to risk. These uses and mixtures of these strategies might therefore depend on both the character of the risk problem (Aven and Renn, 2020) and the conditions of their implementation (Zinn, 2016).

Investigations of PE teachers' RSM practices through their perspectives may increase understanding of PE teachers' risk strategies in practice. This is of vital understanding since it ultimately may have an effect on students' educative and risk experiences in PE. By exploring teachers' RSM practices in PE, this study may contribute to the field of PE practice research by investigating two questions: *what characterizes teachers' risk and safety management practice in physical education*, and *how do teachers relate their practice to risk and safety management?*

## Materials and Methods

A mixed-methods approach was employed in this study as integration of results is believed to add value and a more thorough understanding of the research questions (Creswell, 2015). The data collection was conducted cross-sectionally in Norwegian schools from September to December 2019 through a survey questionnaire and semistructured interviews with teachers. This aim was to generate quantitative data on trends and in-depth qualitative data on the teachers' RSM practices. The construction of the study's instruments was an interactive process; data from each strand of inquiry were analyzed independently, are presented separately in the results section, and thereafter integrated in a discussion to enhance and nuance the results. All language translations from Norwegian into English are made by the first author.

### Survey Questionnaire

#### Participants—Survey

A list of Norwegian primary and lower secondary schools was provided to the researchers by the Norwegian Directorate of Education and Training. In seeking PE teachers who work in public schools who apply the national curricula and regulations for PE, 2,572 schools were contacted to recruit respondents. From the initial number of 949 (*n* = 949) respondents to the survey, 251 (*n* = 251) were excluded because they had not filled any demographic data or finalized the questionnaire by clicking “finish.” The number of respondents included in the analysis was 698 (*n* = 698). As there are no current records of the number of PE teachers in Norwegian schools, additional drop-out statistics are not available. Albeit the sample size is considered to be fairly large (*n* = 698) in a Norwegian PE context, it is not a randomized sample and the results are not generalizable to the population. The respondents still represent all Norwegian counties in 2019 (*n* = 18); they teach in primary (49%), lower secondary (34.1%), and mixed schools including both primary and lower secondary level (16.6%). There is almost an equal gender representation (*m* = 364, *f* = 328) among the respondents. Almost half of the respondents (49.9%) have worked 9 years or less as PE teachers. Beginner teachers comprise 117 respondents (16.8%) who had 2 years or less of PE teaching experience, while 31 respondents (4.4%) had more than 30 years of experience.

#### Instrument Design and Data Collection—Survey

The survey instrument was designed by the researchers as an online questionnaire using *Select Survey* and multiple steps were taken to develop the instrument according to the study's aim and research questions.

First, former research and academic literature on RSM in PE were conferred to gain insight into current knowledge of RSM practices in the field and RSM advice for PE teachers (see, e.g., Young, 2007; Murphy, 2015). Moreover, former PE-related surveys including Norwegian PE teachers were also investigated to inform the design and selection of demographic items (Moen et al., 2018; Statistics Norway, 2019). Statistical literature was also conferred to inform the design and collection of survey data (see, e.g., Ringdal, 2018). An expert in Select Survey at the first author's university was consulted for assistance in the process of coding in the software and in designing the instrument's user interface. This included the visual representation of the survey, information provided to the respondents at different stages of the survey including the definition of RSM used in this study, and sub-explanations to the questions. Using the initial instrument, a small-scale pilot study was conducted and included both PE teachers and PETE educators (*n* = 12). Conversations with representatives from both educator groups as well as opinions on topics and missing and redundant items and values provided information on the face and content validity of the instrument. Their feedback was used to develop and refine the questions and sub-items, and if the clarifying sub-texts were appropriately understood. In addition, based on their feedback about time spent on completing the survey, the survey scope was further adjusted. These steps were then followed by in-depth semistructured interviews with PE teachers (*n* = 17). Data from this study, including the teachers' wording and the topics that were brought up in the conversations, also helped validate the content and in developing and refining the instrument's questions, sub-items, and values to fit with the Norwegian context. The final survey comprised four main topics: *background, experience and opinion, change and development*, and *practice* relevant to this study.

In this article, the following four questions from the survey topic *practice* are reported upon: (1) how often is your risk and safety work part of the following?: teaching preparations, integrated in the teaching, and in the follow-up after teaching (*timing of practice*) on a seven-point Likert-type scale with increasing values from 1 (never) to 7 (always); (2) to what degree are the following elements (shown in Table 1) part of your risk and safety work in PE? (*content of practice*), with eight sub-elements reported on a seven-point Likert-type scale from 1 (not at all) to 7 (always) including the mid-point 4 (neither/nor); (3) describe your risk and safety work by taking a stand to the statements under (shown in Table 2) (*description of practice*), with six statements on a five-point Likert-type scale including the mid-point 3 (neither/nor); and (4) are there any physical activities or teaching methods you exclude from your teaching due to risk for injury and accidents? (*exclusion of activities*), reported on three open response options, whereas the first of the three open response options is presented in this article.

**Table 1:** Content elements given in *content of practice*.

	**Sub-elements**
1	Control and maintenance of equipment and facilities
2	Mapping of risk and danger
3	Developing plans and systems for preventing injuries and accidents
4	Documentation and administration of injuries and accidents
5	Supervision, observation, and overview of students
6	Instruction and guidance of activities
7	Follow-up on rules and routines
8	Facilitation and adaption of activities to the student group

**Table 2:** Statements presented in description of practice.

	**Statements**
1	I mainly use discretion and common sense in this work
2	I mainly use selected method sets in this work
3	The work is mainly based on experiences from teaching
4	The work is mainly based on what I have learned through education and courses
5	The activities I teach determine the way I work
6	The way I work is independent of the activity I teach

### Interviews

#### Participants—Interviews

The interview data include 17 (*n* = 17) primary and lower secondary teachers from eight public schools of different sizes, situated in both rural and city areas in three counties in Norway. A purposeful sampling strategy (Patton, 2015) was applied to select participants. The main criteria for selection was the target group of the study: teachers who teach PE in primary or lower secondary education in the fall of 2019 and in public schools that follow the Norwegian national curriculum and RSM regulations relevant for PE. To obtain study material from teachers with varied characteristics, a second set of criteria was applied: to seek participation from primary and secondary education, both male and female, and participants with a range of age and teaching experience. As the interviews were to be conducted in-person, a pragmatic approach was taken to select teachers from three counties in proximity to the first author's university/work premises. Among the participants, 11 (64.7%) are male and 6 (35.3%) are female. Five teachers (29.4%) work in primary schools, 11 teachers (64.7%) work in lower secondary schools, and one (5.8%) works in a mixed school. The participants had varied educational backgrounds, from no credits in PE teacher education to bachelor's degrees from PE teacher education or physical activity study programs. The participants' ages and PE teaching experience are shown in Table 3.

**Table 3:** Participants' ages and years of PE teaching experience.

Age	20–29	30–39	40–49	50–59	60–69
Total (*n* = 17)	1	4	6	5	1
Years of PE experience	1–5	6–10	11–15	16–20	21+
Total (*n* = 17)	1	3	4	3	6

#### Instrument Design and Data Collection—Interviews

To gain in-depth data on the participants' perspectives on their RSM practice, the first author conducted semistructured interviews with the assistance of an interview guide. Multiple steps were taken to construct the guide according to the research questions and aim of the study. An initial draft was generated based on literature on RSM in PE and the first author's knowledge and experience with RSM. Conversations with PE teacher educators at the first author's university were held about the guide and relevant topics. Conversations with the respondents to the survey pilot study further informed the development and refinement of the guide. The interview guide was designed with main topics, keywords, and some open questions to open for the conversations to evolve and include interesting leads (Gibson, 2010; Patton, 2015). The final guide included six main topics: *background, opinion, societal expectations, change and development, competence and training*, and *practice*. The interviews had a range of 31–69 min and lasted an average of 45 min. All interviews were audiotaped and transcribed verbatim by the first author.

### Data Analysis

This article reports on the survey respondents' demographic data and descriptive statistics of four questions related to RSM practice. IMB SPSS software version 26.0 was used to calculate frequency, percentages, means, and standard deviation on (1) *timing of practice*, (2) *content of practice*, and (3) *description of practice*. The data reported on (4) *exclusion of activities* were categorized with the use of Microsoft Excel by the first author before the categories were cross-checked with the second author and then summarized. The survey results presented in this article are the respondents' assessment and reports on these questions.

Concerning the interviews, the analysis was conducted by the first author. The analytical process began in the interviewing phase and notes were written down during the interviews and while transcribing the material. The coding of the interview material was inspired by a grounded theory approach to emphasize the participants' voices and the empirical data (Charmaz, 2015; Saldaña, 2016). The material was therefore coded *in vivo* line by line (Charmaz, 2015) in the software tool Nvivo 12. These first phase codes were then compared against the full material to look for patterns. Based on significance in the material, a set of focused codes (Saldaña, 2016) were selected in the second cycle phase to construct categories. The following analytical process consisted of memo-writing, interpretation, and checking initial categories against the data and the notes. In the final phase of analysis, three categories anchored in the data were generated—(1) *managing risk in physical education*, (2) *facilitating and modifying physical education*, and (3) *conflicting considerations*—and are presented in the *Results* section.

### Ethical Considerations

For both the survey and the interviews, school management functioned as door openers (Lindsay, 2010) by forwarding e-mails with our requests for participants to PE teachers at their schools. Both participant groups were informed in a cover letter attached to the e-mails about the aim of the study and with a definition of RSM as risk and safety work with the intent to prevent and manage accidents and physical injury to students in PE. They were further informed of their rights and ethical implications and that approval was granted by the Norwegian Centre for Research Data. The participants in the interview study reached out to the first author by e-mail or via their local school management. The interviews were conducted in-person. One of the interviews was conducted at the first author's university by choice of the interviewee and the remaining 16 were conducted on the work premises of the teachers. The participants were again informed of the study's aim and asked if they had any questions before they signed a consent form and audio taping was approved by each teacher before the interviews began. Directly identifiable data were deleted in transcribing the conversations. With regard to the survey, the respondents were informed of the procedure that they gave their consent by answering the survey and clicking “finish” on the last page. After the responses were downloaded to a secure location, the online survey with the results was deleted. Although the two participant groups in this article are independent of each other, the interview participants may have responded to the survey as it was distributed to their school management as they were among the schools (*n* = 2,572) that were contacted by e-mail with information of the study and a link to the survey.

## Results

The survey and interview results are presented separately in succeeding sections. These include reports on the four survey questions: (1) *timing of practice*, (2) *content of practice*, (3) *description of practice*, and (4) *exclusion of activities* and three categories from analysis of interviews: (1) *managing risk in physical education*, (2) *facilitating and modifying physical education*, and (3) *conflicting considerations*.

### Results From the Survey

#### Timing of Practice

To explore when, during their work days in relation to teaching PE classes, teachers practice RSM, the respondents were asked how often their risk and safety work is part of the teaching preparations, integrated in the teaching, and in the follow-up of PE classes.

**Figure 1:**
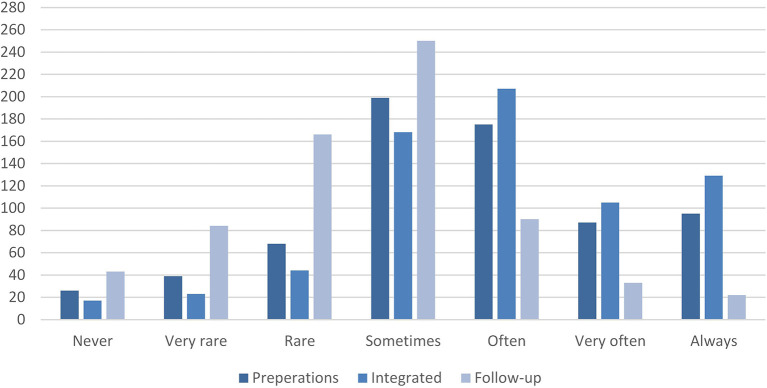
Teachers' reports on the *timing of practice* (by frequency).

Table 4 shows that the majority of the respondents report that risk and safety work are sometimes or more frequently part of the preparations (*M* = 4.6, *SD* = 1.521). However, only 13.6% report that RSM is always part of the preparations. Then, again, there are also teachers (3.7%) who never include RSM as part of their preparations. A picture of RSM as an integrated practice is drawn as it is reported to be even more frequently integrated in the teaching of PE classes (*M* = 4.96, *SD* = 1.444). There are more respondents who report that RSM is always (18.5%) or very often (15.0%) integrated in the teaching than in the preparations. The results here, on the other hand, differ for RSM as part of follow-up in teaching PE classes, as fewer respondents report that RSM is often, very often, or always part of the follow-up (*M* = 3.65, *SD* = 1.335). To provide an illustration of teachers' reports on the *timing of practice*, Figure 1 shows the distribution of teachers' responses.

**Table 4:** Teachers' report on how often RSM is part of the preparations, integrated in teaching, and in the follow-up of PE classes (by percent).

	**Never**	**Very rare**	**Rare**	**Sometimes**	**Often**	**Very often**	**Always**
Preparations (*n* = 689)	3.7	5.6	9.7	28.5	25.1	12.5	13.6
Integrated (*n* = 693)	2.4	3.3	6.3	24.1	29.7	15.0	18.5
Follow-up (*n* = 688)	6.2	12.0	23.8	35.8	12.9	4.7	3.2

The results shown in Figure 1 illustrate the difference in the reported frequency of RSM as part of follow-up compared to preparations and integrated in teaching. Teachers may conduct RSM at different times in relation to teaching, and it might also depend on the content of, their RSM practices.

#### Content of Practice

To gain insight into what teachers do in terms of the content of teachers' RSM practices, the respondents were asked to what degree eight content sub-elements (shown in Table 1) are part of their risk and safety work.

The results shown in Table 5 suggest that the content of teachers' RSM practice tends toward teaching active content elements including instructing activities (*M* = 5.99, *SD* = 0.981), supervising students (*M* = 5.96, *SD* = 1.054), facilitating activities for the students (*M* = 5.84, *SD* = 0.933), and follow-up on rules (*M* = 5.74, *SD* = 1.089). The elements that are more remote from the active teaching, especially developing plans and systems (*M* = 3.68, *SD* = 1.534), are to a lesser degree reported to be in the content of teachers' RSM practice. To gain a more comprehensive picture of teachers' RSM practices, the respondents were asked to describe their RSM.

**Table 5:** To what degree are the following elements part of your risk and safety work?

	**Mean**	***SD***
Control and maintenance of equipment and facilities (*n* = 691)	4.36	1.496
Mapping of risk and danger (*n* = 682)	4.96	1.366
Developing plans and systems for preventing injuries and accidents (*n* = 681)	3.68	1.534
Documentation and administration of injuries and accidents (*n* = 685)	4.61	1.760
Supervision, observation, and overview of students (*n* = 694)	5.96	1.054
Instruction and guidance of activities (*n* = 689)	5.99	0.981
Follow-up on rules and routines (*n* = 685)	5.74	1.089
Facilitation and adaption of activities to the student group (*n* = 691)	5.84	0.933

#### Description of Practice

When the teachers were asked to describe their risk and safety work by taking a stand on the six statements shown in Table 2, the results in Table 6 show that teachers' use of discretion and common sense is reported to be higher in agreement (*M* = 4.51, *SD* = 0.708) than teachers' use of selected method sets (*M* = 3.46, *SD* = 1.024). In more detail, most respondents (93.1%) slightly or completely agree with the use of discretion and common sense in their RSM. This is interesting compared with the use of selected method sets, where quite a few seem to neither agree nor disagree (36.5%), but there are still quite a few who slightly agree (29.8%). This general picture seems to match teachers' agreement of RSM based on experience (*M* = 4.34, *SD* = 0.757) rather than RSM as learned from education and courses (*M* = 3.67, *SD* = 1.106). Most respondents (88.9%) slightly or completely agree that RSM is based on experience from teaching, and there are more teachers (14.7%) that completely or slightly disagree that their RSM is mainly based on education and courses. In addition, more teachers seem to agree about the activities taught to determine the (RSM) work (*M* = 4.49, *SD* = 0.691) rather than being a more general approach reported as work independent of activities taught (*M* = 2.69, *SD* = 1.365). Regarding the activities taught to determine teachers' RSM, most respondents (91.8%) slightly or completely agree to this statement. However, these reports fairly coincide with (RSM) work as independent of activities taught, as approximately half of the respondents (48%) slightly or completely disagree.

**Table 6:** Describe your risk and safety work by taking a stand on the statements below.

	**Mean**	***SD***
I mainly use discretion and common sense in this work (*n* = 693)	4.51	0.708
I mainly use selected method sets in this work (*n* = 678)	3.46	1.024
The work is mainly based on experiences from teaching (*n* = 689)	4.34	0.757
The work is mainly based on what I have learned through education and courses (*n* = 684)	3.67	1.106
The activities I teach determine the way I work (*n* = 689)	4.49	0.691
The way I work is independent of the activity I teach (*n* = 683)	2.69	1.365

#### Exclusion of Activities

As the PE literature points to risk aversion and considering that teachers' RSM practices might relate to the physical activities taught in PE classes, the respondents were asked if there were any activities or teaching methods they excluded from their PE teaching due to the risk of injury and accidents. In reporting on the potential activities or teaching methods that teachers excluded from PE, Table 7 shows the results from the respondents' first response in a rank from 1 to 5 (6).

**Table 7:** Teachers' report on exclusion of activities by percent (*n* = 238).

**Rank**	**Activity**	**Percent**
1	Trampoline gymnastics	26.8
2	Gymnastics general	18.0
3 (split)	Rotations gymnastics	7.5
3 (split)	Climbing activities	7.5
4	Contact sports	7.1
5	Outdoor swimming	6.3

Table 7 highlights what kind of physical activities are excluded due to risk. The reported excluded activities that are connected to gymnastics as a whole (trampoline, rotations, and gymnastics in general) make up 52.3% of the responses.

### Results From the Interviews

The following three categories from the interviews with teachers are presented in the next sections: (1) *managing risk in physical education*, (2) *facilitating and modifying physical education*, and (3) *conflicting considerations*. The excerpts selected serve as examples of category meaning. The codes used in the excerpts (e.g., IP3) are based on Interview Person and a number given to each participant.

#### Managing Risk in Physical Education

Teachers in this study initially express a general lack of RSM practice, and they are divided in their explicit use of any risk measures and in the attention they give to RSM in PE. However, when they are asked to describe their RSM practices, the participants consistently begin with and bring up water activities and gymnastics where they apply RSM plans or procedures to their teaching practices. This includes, for example, safety principles for spotting jumps on trampolines or organizing lessons in water in a specific manner.

We took a course [in outdoor swimming], and we think about safety a lot, how we are to conduct it for those who are on land and those who are in the water. We never have groups larger than eight at the same time, we always have two [teachers], one teaches and one watches, there are two more [teachers] on land who guard those [students] on land, and we make boundaries for the area in the water with ropes and on land with cones (IP3).

Hence, the part of the teachers' RSM practice that are drawn on procedures are commonly applied to physical activities that are perceived to be riskier or more dangerous for the participants in this study. However, the teachers still describe limited system approaches for RSM in PE in their teaching and in their schools. Exceptions are made for a few externally mandated requirements in certain areas of PE as they are compelled to file reports following student injuries and partake in an annual safety inspection of equipment, materials, and facilities. The system of controlling and documenting potential deficits might be ceremonial to teachers because

if [the equipment] is not replaced, it has at least been recorded in a document (IP14).

Against the backdrop of these formalized RSM requirements, field trips, and sporting and recreational events signify a shared customary routine established among the teachers in their schools. They

talk about it every year, who should walk in front, who should walk behind [the group] and who should carry the emergency kit (IP14).

However, the material suggests that PE teachers worry about the pulverization/shredding of responsibility for these events because of the number of teachers involved and they personally are not necessarily in the lead or in control of what happens. The way teachers perceive or frame RSM in PE is central to the results because an impression is made of RSM as comprising of procedural and formalized measures for risk control. Considering that RSM might be a formal practice to these participants, their practice that are pertinent to other pedagogical concerns might still make an essential contribution to their RSM practice.

#### Facilitating and Modifying Physical Education

As their framing of RSM might be oriented toward plans and procedures, RSM is portrayed as difficult or even meaningless to implement in PE in some respects. Regarding RSM training, a participant does not seem to find it applicable for PE, and as a result,

it would be hard to be trained. I do not see what it should have been—gymnastics yes, because then your back and neck are central, but in most other activities the whole body is used, in a different way.… It would have been terribly interesting. I wonder what that course would have been like if let us say you have ball activities, dance or outdoor education (IP17).

As indicated in the above excerpt, the participant includes gymnastics as appropriate for a defined risk strategy while excluding other physical activities to fit the same approach. When the participants are asked to go into more detail about their teaching, they talk about teaching principles and pedagogical and didactical deliberations. Mastery, students succeeding, learning, and having fun in PE classes are given as reasons for adjusting and modifying the program, and not risk.

During the conversations, however, the teachers in this study became more verbal and explicit in connecting this part of their teaching practice to risk concerns. These incorporate adaptations of the curriculum, selecting, and adjusting physical activities and sports to the teachers' preferences and the local context, including modifying the activities taught in the PE program to fit with the students' characteristics. The teachers talk of both the need to reduce risk in some respects and the need to facilitate students' learning. Combining strategies is performed by conducting both risk analysis and modifications to physical activities as indicated by a participant in the next excerpts.

One must always perform a risk analysis. In natural science, for example, following the principle that if you do an experiment you must do it at the lowest level possible, you do not start with hydrochloric acid if citric acid does the same trick. I think in the same way about PE; you must consider what you want to achieve, and then you must take risk into the whole picture if something can happen (IP1).

It is not a homogenic group. They are not athletes. There are people of 40 and 100 kilos. It is rare that you see those gatherings [except in PE]. You must always choose physical activities that you can adapt to all (IP1).

The modifications to the program are thus made in accordance with the students' characteristics at an appropriate level of difficulty or variant of physical activities taught. Knowing the students well, therefore, seems to be key for teachers' opportunities to make these adjustments in the programs.

I see and observe and feel and talk with the students and determine their limits, each single student in a way (IP10).

For teachers to facilitate and adapt their PE classes to the students' characteristics and needs, knowing the students is of utmost importance. Part of the teachers' RSM practice involves the students in risk judgments and makes students responsible for their behavior. A participant also had a strategy of making students feel responsible for what might happen in class by telling them of unsuccessful stories and about the responsibilities of teaching. This includes talking about possible scenarios, what is important for safety in PE and teaching students how to behave toward others. Verbal reminders and addressing students' consciousness and conscience seem to be part of teachers' RSM practices. Establishing rules of conduct is also an appreciated strategy among teachers because students seem to respect the rules when they know of the potential risks involved. Thus, two branches of strategies seem to develop and incorporate teaching students how to be responsible and then make them aware of teachers' responsibilities and the risks involved. Teachers portray this to be part of students' character building and learning in PE and argue that it is part of teachers' mandate and the curriculum. A reference to outdoor education is made to explain the logic.

As a teacher, I must have enough knowledge about it to teach students to understand that they are also resources, that we have to take active responsibility during outdoor field excursions, be a resource. Students need to be aware that when they go on their own excursions, they have to be the person who would have to save their friend (IP1).

There is also a strategy of protection of girls from a grouping of rough boys which is the reason for separating genders in the data. It is not used as a general or permanent solution among the teachers, but rather,

in some of the activities, it is a part of the preparations to ask whether they would rather play girls against girls and boys against boys (IP6).

Risk is in one sense dealt with by separation of students, but how cautious boys are considered is not clarified. Rough student groupings might induce teachers to protect other students in PE programs; indeed, there is no addressing of managing the boys' roughness in the data. Regarding dangerous physical activities, however, exclusion seems relevant as a measure.

You might cut it out [of the PE program] completely, instead of doing it with a safeguard, you exclude things (IP15).

Teachers may therefore eliminate risks by exclusion rather than adapting or modifying the activities taught. This strategy is contended among the teachers due to educative concerns. Although they experience gymnastics to induce risk, some still choose to include it because exclusion might deprive students' development, especially skilled gymnasts. Risk seems therefore to generate conflicting considerations for teachers in PE.

#### Conflicting Considerations

Teachers may exclude some physical activities, and there is also resistance toward risk-averse strategies among the participants in this study. In choosing among different strategies, it may, in some respects, for these teachers, manifest itself as a choice between restricting students or accepting risk of injury. Although the participant might be aware of the risk involved, other pedagogical concerns seem to triumph.

Often there is a need for splitting the class into groups and to do activities in two halls, and I cannot be in two places at the same time, but [I] still choose to organize the activity in a way that makes it possible to do different things (IP16).

Teachers seem torn between educational considerations and the potential adverse consequences of risk. The tension is clear for the participant:

If we want to implement some things, it requires a [particular] organization; the risk lives its own life, and then you are in danger of regretting it bitterly if anything happens (IP16).

The results are further discussed in the next section, and Zinn's (2016) risk strategy typology is applied to distinguish among teachers' strategies and suggest how teachers combine them in their practices.

## Discussion

By combining the quantitative and qualitative data and applying the lens of Zinn's (2016) risk strategy typology to teachers' practices, the article seeks to discuss the two research questions: *what characterizes teachers' risk and safety management practice in physical education* and *how do teachers relate their practice to risk and safety management*?

The results in this study suggest that teachers' RSM practices are multifaceted. The interviewees initially describe their RSM practices as quite scarce. This might contrast a former Canadian study (Young, 2007, 230) where teachers claim that risk management is vital in their PE planning and teaching. During the conversations, however, the teachers are more explicit in connecting their adaptations and facilitative measures to RSM. As a result, their RSM practices seem far more comprehensive and the strategies that are embedded into the teaching might make out the greatest contribution in their RSM practices. Based on these results, it is expected that the use of rational risk strategies that explicitly target risk (Zinn, 2016), such as risk matrices or other risk-analytical instruments, might be less prominent in teachers' RSM practices. However, the respondents in the survey report that this type of strategy, such as mapping risk and danger (*M* = 4.96), make a generous part of their practices. Therefore, the survey results seem to diverge from the interview results in some respects and for which might nuance the first impression. On the other hand, developing plans and systems for preventing injuries and accidents (*M* = 3.68) is reported to be the least part of the respondents' RSM practices among the categories presented to them (Table 5). This converge with the interviewees' report on a lack of systems approaches in PE. It seems that teachers' RSM practices are selective and that teachers may apply such strategies and not necessarily create systems for risk in PE.

What comprise the most central contributions to teachers' RSM practices, reported in *content of practice* in the survey, are instruction and guidance of activities (*M* = 5.99) and supervision, observation, and overview of students (*M* = 5.96), which may signal strategies that are integrated into teaching. Moreover, when the survey respondents are asked to describe their risk and safety work, they greatly agree that they use discretion and common sense (*M* = 4.51) and that their practices are based on teaching experience (*M* = 4.34) seen in Table 6. They agree less to draw on what they have learned through education and courses (*M* = 3.67) and the use of selected method sets (*M* = 3.46). These survey results seem to converge with the interviews: teachers mainly talk about experience and practical know-how in their practice (Polanyi, 1983; Schön, 1995). Hence, looking into teachers' *timing of practice* (Table 4), an abundance of the respondents in the survey (87.3%) report that RSM is a practice sometimes or more frequently integrated in teaching, and a great deal (79.7%) report that RSM is sometimes or more frequently part of their teaching preparations. This may therefore characterize teachers' RSM practices as flexible and with reactive measures being slightly more prominent. The results here might signify that ongoing judgment and action during teaching is vital for teachers.

Then, again, exclusion might be a common risk strategy in PE as teachers in this study do exclude physical activities due to risk. The activities that were mentioned in the survey (Table 7) and in the interviews are converging. Still, as more than 50% of the responses relate to gymnastics, it seems to be a limited practice. However, risk practices are embedded within institutional and cultural environments (Douglas, 1992; Lupton, 2013). In Park's (2018) Korean study, specialist PE teachers report that they are hesitant to teach physical activities that are accident-prone due to safety policies. Among the interviewees in this study, policy is not given as a primary reason for exclusion of physical activities, but it more so relates to safety concerns such as in Canada (Young, 2007). Hence, it might be that teachers' interpretations of PE and the curriculum promote exclusion as a risk strategy in Norway.

How teachers relate their practice to RSM is therefore central to the investigations and the results in this article. One potential explanation to the interviewees' initial descriptions of RSM as scarce might be found in their framing of RSM and the rationales behind different strategies (Zinn, 2016). The material indicates that RSM is framed by the interviewees as a formal and explicit risk practice. Prescribed plans and procedures for dealing with risk are thus central and seem to draw on an instrumental risk management logic (Zinn, 2016). In consequence, these teachers do not necessarily conceptualize some of the RSM content described in the survey as RSM but rather measures of other pedagogical concerns. Considering the adaptations and modifications teachers make to program activities, they may correspond to in-between risk strategies (Zinn, 2016) with an essential function in PE: it might be the part of teachers' RSM practices that cater to uncertain risk (Renn, 2008; Aven and Renn, 2020). These measures might make a fluid transition between in-between and rational strategies in PE. The use of rational strategies, as a consequence of being more abstract approaches (Zinn, 2016), might fit with the measurable and controllable dimensions of risk (Renn, 2008), and for some teachers, it is limited to injury reporting and annual inspections of facilities. An interpretation can be that this relates to accountability and school safety policy such as in Korea (Park, 2018), as reporting may relieve the participants' or schools' risk-related liability. An issue that may arise with some of these strategies, however, similar to insurance and assurance, is that they do not necessarily deal with the risk of injury but the potential negative outcome—to maintain trust among stakeholders and give an impression that students' safety is secured (Lindqvist et al., 2009). It may also suggest that their explicit risk practice might include a rationale of risk control that is not necessarily applicable to all areas of PE. Although it may be beneficial in documenting default equipment or accidents that have happened, these strategies might not fit areas of uncertainty or pedagogical concerns of teaching. In outdoor adventure, it is the complex and flexible character of the work that is highlighted in research (Collins and Collins, 2013; Mees et al., 2020). Against this backdrop, the case of compulsory safety guidelines seems questionable. While imposing peer controls and making teachers accountable for applying procedures and guidelines might be beneficial in some situations (Fitzgerald and Deutsch, 2016), it may also restrict teachers' RSM practices to a practice that includes mainly rational strategies. This may be problematic as risk models and procedures might give a false impression of safety in some respects—albeit rational, they might also be questionable (Cox, 2008).

In teaching physical activities that entail a potential for severe injury such as swimming and gymnastics, teachers make use of preplanned procedures that seem to correspond to rational strategies (Zinn, 2016). Although teachers also relate their use of safety guidelines to liability internationally, the target activities and risk-severity reasons in this study seem to match international reports from PE (Young, 2007; Rothe, 2009; Park, 2018). Considering safety procedures that are applied to, for example, outdoor swimming, the knowledge basis for these procedures, however, suggests that formal training is important for teachers to gain knowledge of and to include rational strategies in their RSM practices and potentially manage severe risk in PE. Teacher education and teachers' professional learning opportunities might therefore have an important function in complimenting teachers' use of in-between strategies in their RSM practice.

The participants in the interview study also talk about exclusion in relation to something they dread that resonates with emotions as an in-between strategy to deal with risk (Lupton, 2013; Zinn, 2016). It might be further understood as teachers enact the precautionary principle and attempts to eliminate unnecessary risk (van Asselt and Vos, 2006; Zinn, 2016). Nonetheless, there is a potential for inclusion of risky activities if they are vital for students' development as both the results from the survey and the interviews suggest that there is space for teachers to develop their formal practices and implement rational strategies to a greater degree. It is therefore important to support teachers in developing RSM strategies for the activities they find challenging, especially if they are compulsory curricular activities. The curricular framework for PE in Norway and teachers' interpretation and performance of PE might be apparent in teachers' RSM concerns in practice. Their concerns about gymnastics, for example, might suggest that teachers identify the activity as a central component in PE. Enforcing activities in which teachers are not competent is a policy and management responsibility. Considering that teachers apply plans and procedures to some risky activities, exclusion might be a reasonable strategy given a lack of rational means and in contrast to passive risk acceptance (Zinn, 2016). Professional self-regulation and teachers adjusting their practices by not including activities in which they are not familiar and competent may compliment a reasonable RSM practice (Zinn, 2016). On the other hand, exclusion practices might be problematic if students are included. One example might be illustrated by student management. An impression is made that the interviewees might deal with the risk that is generated by rough boys by physically separating the girls. Another Norwegian study from 2019 also suggests that outdoor education teachers exclude some students from excursions as a safety strategy (Dahl et al., 2019).

Teachers' RSM practices might include a willful lack of risk control anchored in their pedagogy. However, educative uncertainty practices in PE (Quennerstedt, 2019) might create value ambiguity (Aven and Renn, 2020) among teachers. In an environment that promotes uncertainty, it might be difficult for teachers to induce an all-safety-first teaching practice if it deprives students of learning opportunities. A controversy therefore seems to arise in relation to teachers' inaction to engage in rough behavior in this study and whether this relates to passive or active risk acceptance (Zinn, 2016, 2019). A question remains for teachers: Do they have the means to manage the rough group, or do they accept the behavior due to pedagogical or other preferences? A potential explanation might be that risk-reducing measures may affect other pedagogical aspects of the program. In controlling activities when teaching gymnastics, for example, teachers may single out the individual performers in a negative way. The alternatives for teachers might be to either generate or accept risk to enhance student learning or reduce or otherwise eliminate risk and students' opportunities. With this purpose, risk generation or acceptance might include a non-rational strategy of “wishful thinking” or hope in that an accident will not happen (Zinn, 2016).

In teachers' institutionally framed mandate of school safety, however, it might not be appropriate for teachers to rely on hope, as societal norms and rationales for risk strategies are culturally, institutionally and situationally dependent (Douglas, 1992; Lupton, 2013; Zinn, 2019). Conflict of understanding may lead to teachers excluding activities such as in Korea (Park, 2018). It may be troublesome that some teachers apply caution and even exclude certain physical activities, while others might embrace risk. Different practices and norms among teachers may cause tension and divergence in the field of practice in Norway. However, tension in professionals' work with risk seems to be common (Brown and Gale, 2018b). The practice also depends on school policy: if stakeholders support a risk–benefit balance, there must be an equal acceptance that accidents may happen. These Norwegian teachers' institutional environment, however, seems to open for a mixture of strategies (Zinn, 2016, 350). When teachers experience conflicting considerations on the other hand, the curricular framework and safety regulations might not align, which is clearly of relevance when practicing and policing RSM in PE.

## Conclusions

Based on the reports from the survey and interviews with teachers, the results suggest that teachers apply and combine multiple strategies with differing risk rationales. Albeit the interviewees may employ preplanned procedures, the results therefrom still indicate that the use of these strategies is limited. However, the survey results suggest otherwise and therefore diverge in some respects. The survey respondents report that plans and procedures and mapping of risk and danger, for example, to be generous contributions in their RSM practices. Central in the results from both the survey and interviews are the predominance of discretion and activity-based measures in teachers' RSM practice. Albeit teachers make modifications and facilitate the program for students, the interview participants do not necessarily conceptualize this part of their practice as RSM. Overall, measures that initially meant to cater to other pedagogical concerns seem to be vital contributions in teachers' RSM practices. However, conflicting considerations might create tension. While teachers' pedagogies may influence teachers to accept risk for educative reasons, safety concerns might influence teachers to exclude certain physical activities from PE.

The aim of this article was to explore and understand teachers' RSM practices through investigating two research questions: *what characterizes teachers' risk and safety management practice in physical education* and *how do teachers relate their practice to risk and safety management*? The multiplicity of concerns and use or combination of risk strategies in teachers' RSM practices presented in this article seem to constitute a complex endeavor. The ways teachers seem to combine different strands of strategy in dealing with risk in addition to other pedagogical considerations in PE might both deviate from and nuance the promotion of what seems to be rational risk strategies in the PE literature on RSM. It more so seems to be a flexible balancing act that resonates with RSM of other professions (Horlick-Jones, 2005; Collins and Collins, 2013; Brown and Gale, 2018a,b; Mees et al., 2020), which shows the importance of gaining teachers' perspectives on practice. The results also suggest that combining quantitative and qualitative data is fruitful to gain knowledge of the complex character and nuance teachers' RSM practices. The data in this study might enhance the current state of knowledge and contribute to PE practice research. Overall, this may create a stronger foundation for developing RSM practice, theory, and policy in the field of PE.

The research that is undertaken is still with limitations and there are multiple avenues for further research to generate knowledge of teachers' RSM practices in PE. As this study is conducted in a Norwegian PE framework, there might be both similarities and potential differences between teachers' RSM concerns of practices in an international context. It is necessary to consider the Norwegian context of PE including the national curriculum and teachers' performance of the curricula. Although the study provides data from both interviews with teachers and survey results on teachers' RSM practices, observation studies may complement, and add to teachers' practice perspectives. To what extent it is possible to prevent incidents, accidents, and injuries while securing students' educative opportunities if teachers made changes to their RSM practices remains uncertain. In addition, giving voice to the students' perspectives on RSM in PE and teachers' practices are relevant for developing RSM but also theory grounded in the students' perspectives. Thus, how teachers may develop their RSM practices to deal with risk and uncertainty in PE seems relevant for intervention studies. Shedding light on teachers' institutional environments and how they influence their RSM practices in PE seems equally relevant, as risk practices are not constructed or performed in vacuum.

## Data Availability Statement

The datasets presented in this article are not readily available because the data cannot be shared for privacy restrictions. Requests to access the datasets should be directed to lise.porsanger@ntnu.no.

## Ethics Statement

The studies involving human participants were reviewed and approved by Norwegian Centre for Research Data. The patients/participants provided their written informed consent to participate in this study.

## Author Contributions

LP designed the study, performed the data collection, analyzed the data, and wrote the manuscript. LM contributed to the design, to the analysis of the survey results, and to the writing of the manuscript. All authors contributed to the article and approved the submitted version.

## Conflict of Interest

The authors declare that the research was conducted in the absence of any commercial or financial relationships that could be construed as a potential conflict of interest.

## Abbreviations

PE: physical education
RSM: risk and safety management.
